# Constructing Polymetallic Nodes in Metal–Organic Frameworks Enhance Antibacterial of Drug‐Resistant Bacteria

**DOI:** 10.1002/advs.202501327

**Published:** 2025-04-26

**Authors:** Qinqin Li, Shihan Zhang, Yachao Xu, Yaru Guo, Youxing Liu

**Affiliations:** ^1^ Beijing Key Laboratory of Electrochemical Process and Technology for Materials Beijing University of Chemical Technology Beijing 100029 China; ^2^ Department of Geriatric Dentistry Peking University School and Hospital of Stomatology Beijing 100081 China; ^3^ School of Materials Science and Engineering Peking University Beijing 100871 China

**Keywords:** antibacterial, antibacterial therapy, metal–organic framework materials, polymetallic coordination node, reactive oxygen species

## Abstract

The misuse of antibiotics results in the emergence of a large number of drug‐resistant bacteria, which leads to huge financial and social burdens. Exploring artificial nanozymes is regarded as a promising candidates for the substitution of antibiotics, but still remain a huge challenge. Herein, a new strategy is reported for constructing polymetallic indium coordination node Metal‐organic frameworks (MOFs) (polyIn‐BTB) for enhancing the production of reactive oxygen species (ROS), which significantly promote antibacterial activity. Theoretical research reveals that, compared to monometallic indium coordination node MOFs (monoIn‐BTB), polyIn‐BTB exhibits a stronger electron‐donating ability, which can facilitate the efficient production of ROS. Thus, polyIn‐BTB shows outstanding antibacterial properties of 87.0% and 92.0% for Methicillin‐Resistant Staphylococcus aureus (*MRS. aureus*) and *Escherichia coli* (*E. coli*) respectively, which is significantly higher than that of monoIn‐BTB (42% for *MRS. Aureus* and 50% for *E. coli*). The in vivo experiments demonstrate that polyIn‐BTB accelerates wound healing by killing bacteria and inhibiting the inflammatory response they cause, with a wound healing rate of 98.0% in 8 days. Overall, this work reports a new strategy for constructing polyIn‐BTB for enhancing the antibacterial performance, which opens the door to fundamental research on designing the nanozyme with high performance.

## Graphical abstract

This work reports a new strategy for constructing multi‐nuclear metal coordination nodes in MOFs for boosting photocatalytic ROS production, which significantly enhances the antibacterial efficiency relative to the MOFs with single‐nuclear metal coordination nodes.

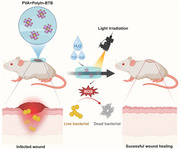

## 1 Introduction

Bacterial infections have been considered to pose a significant challenge to global public health, causing illness and death for millions of people each year.^[^ ^1^, ^2^ ^]^ These infections are prevalent in hospitals, medical facilities, food production, and natural environments.^[^ ^3^, ^4^ ^]^ The extensive and diverse nature of bacteria makes them capable of causing various diseases, such as pneumonia, skin infections, and food poisoning. Since the discovery of penicillin by Alexander Fleming in 1928, antibiotics have been widely used for antibacterial treatments.^[^ ^5^ ^]^ However, the misuse of antibiotics has led to the widespread emergence of antibiotic‐resistant bacteria, which are increasingly resistant to traditional antibiotics, making treatments more difficult and expensive and placing a significant burden on public health systems.^[^ ^6^, ^7^ ^]^ Biological enzyme technology is one of the promising candidates to substitute antibiotics for antibacterial purposes, but it suffers from various bottlenecks, such as expensive costs, sensitivity to the environment, and non‐reusability.^[^ ^8^ ^]^ In recent years, nanozymes have currently a hot research topic as the candidate of biological enzymes for antibacterial by light‐driving production of reactive oxygen species (ROS), including hydrogen peroxide (H_2_O_2_), hydroperoxy (OH) and superoxide radicals(·OOH), from O_2_ or H_2_O as the initial reagents.^[^ ^9^, ^10^ ^]^ ROS can rapidly damage bacterial cell membranes and cell walls, achieving efficient sterilization and demonstrating broad‐spectrum antimicrobial effects against various microorganisms.^[^ ^11^, ^12^ ^]^ Meanwhile, ROS ultimately decomposes into water and oxygen, which is one of the environmentally friendly antibacterial methods.^[^ ^13^ ^]^ Various nanozymes materials, including metallic ion materials, organic materials, natural antibacterial materials, and semiconductor photocatalytic materials, have been developed and applied for antibacterial applications,^[^ ^14^, ^15^, ^16^ ^]^ but still have notable drawbacks, such as short usage times, high doses, and rapid release of active substances.^[^ ^17^ ^]^ Therefore, it is urgent to explore nanozyme materials with highly efficient, low‐dose, long‐lasting antibacterial.

Metal–organic frameworks (MOFs) are a class of coordination polymers formed by the self‐assembly of metal ions (or metal clusters) and organic ligands.^[^ ^18^, ^19^ ^]^ Due to their high specific surface area, abundant metal nodes, and tunable porous structure, MOFs have been widely used in areas such as gas adsorption and separation, sensing, catalysis, and electrochemistry.^[^ ^20^, ^21^, ^22^ ^]^ In recent years, their potential in biomedical applications, including drug delivery, biosensing, wound healing, and antibacterial uses, has been significantly expanded due to their structural diversity, low toxicity, and good biocompatibility.^[^ ^23^, ^24^ ^]^ The antibacterial performance of traditional monometallic‐node MOFs (e.g., Ag‐, Cu‐, Co‐, and Zn‐based materials) is often limited by the inherent defects of their single active sites.^[^ ^25^, ^26^, ^27^ ^]^ The “point‐point” single metal site adsorption mechanism of monometallic nodes is not conducive to the adsorption of O_2_ or H_2_O molecules,^[^ ^28^, ^29^, ^30^ ^]^ resulting in low ROS generation efficiency. To address these challenges, we speculate that constructing polymetallic MOFs with unique advantages, such as high electron density and stronger electron‐donating ability, can enhance ROS production for improving the antibacterial activity,^[^ ^31^, ^32^, ^33^, ^34^ ^]^ but has not been explored yet.

**Figure 1:**
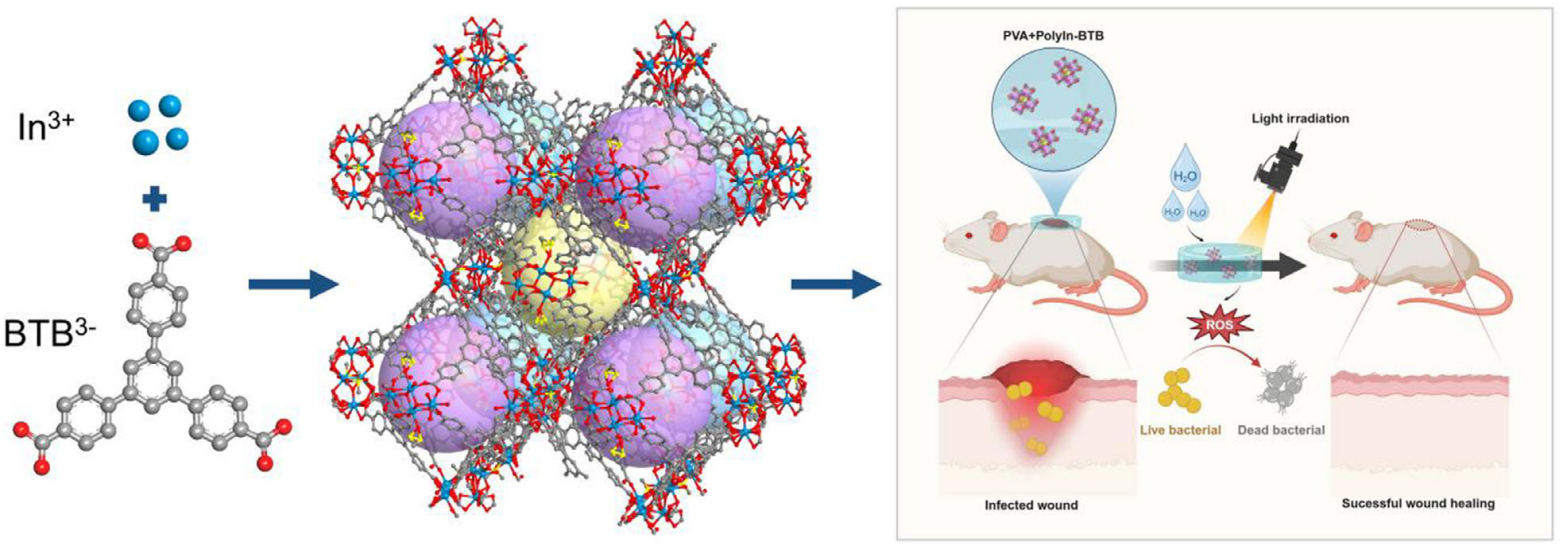
Schematic illustration of polyIn‐BTB structure for antibacterial.

Herein, we developed a new class of polymetallic In coordination node MOFs (polyIn‐BTB) by using H_3_BTB (1,3,5‐tri(4‐carboxyphenyl)benzene) as the organic ligand (**Figure** **1**). The density functional theory (DFT) theoretical calculation shows that polymetallic metal nodes can increase the electron density of MOFs, which is beneficial for the adsorption of O_2_ and H_2_O for accelerating the photosynthesis of ROS for enhancing the antibacterial of drug‐resistant bacteria. In vitro antibacterial experiments show that polyIn‐BTB exhibits the antibacterial rate of 87.0% and 92.0% for Methicillin‐Resistant *Staphylococcus aureus* (*MRS. aureus*) and *Escherichia Coli* (*E. coli*) respectively, which is significantly higher than that of monoIn‐BTB (42% for *MRS. aureus* and 50% for *E. coli*). In vivo antibacterial experiments show that polyIn‐BTB has a more effective wound skin cure rate than monoIn‐BTB, reaching 98.0% of the wound closure rate in 8 days (Figure 1).

## 2 Results and Discussion

### 2.1 Characterization of polyIn‐BTB and monoIn‐BTB

**Figure 2:**
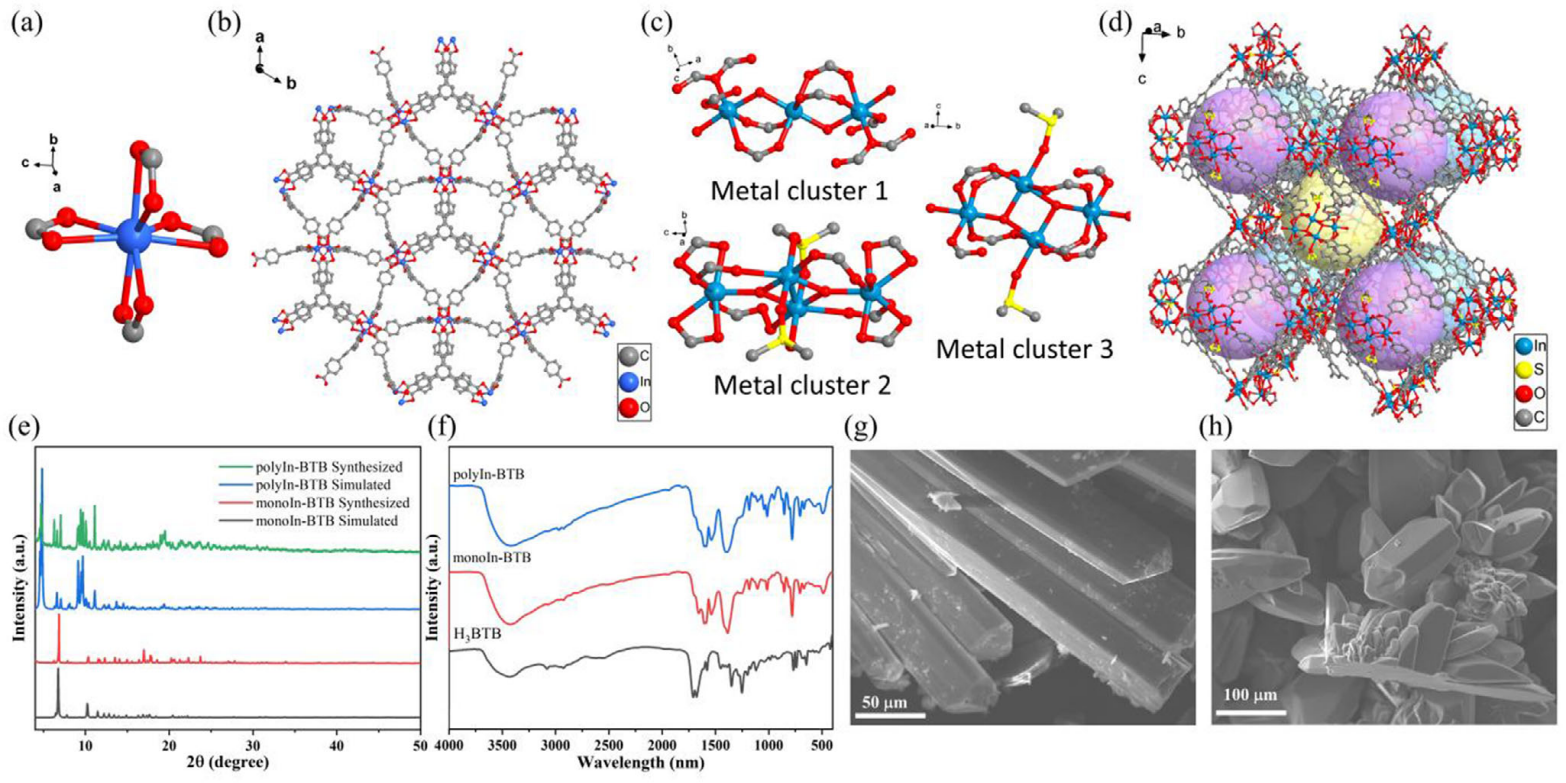
a) The coordination environment of In^3+^ ion in monoIn‐BTB. b) The 3D structure of monoIn‐BTB along the *c*‐axis. c) The coordination environment of three different metal clusters in polyIn‐BTB. d) The 3D structure of polyIn‐BTB along the *a*‐axis. e) XRD patterns of monoIn‐BTB and polyIn‐BTB. f) FT‐IR spectra of H_3_BTB, monoIn‐BTB, and polyIn‐BTB. g) SEM image of a monoIn‐BTB crystal. h) SEM image of polyIn‐BTB crystal.

The monoIn‐BTB crystallizes in the space group *P* $\bar{6}$2*c* and is similar to the reported one.^[^ ^35^ ^]^ The asymmetric unit contains two‐thirds BTB^3−^, a half In^3+^ ions, and a half [NH_2_(CH_3_)_2_]^+^ ions (Figure S1, Supporting Information). There are two conformation BTB ligands, the benzene ring locate in one plane is located in one plane, which is parallel to the *ab* plane, named BTB‐2, and the benzene ring not located in the same plane named BTB‐1 (Figure S2, Supporting Information). Each In^3+^ ion is coordinated by eight carboxylic oxygens from three BTB‐1 ligands and one BTB‐2 ligand (**Figure** **2**a). Each carboxyl group of BTB ligand chelated an In^3+^ ion. Among them, BTB‐1 forms In^3+^ by connecting carboxylic acids in three directions, respectively, which further forms 2D layers (Figure S3, Supporting Information). The 2D layers with three orientations interweave and further interconnect through BTB‐2 chelating In^3+^ ions, resulting in a 3D structure (Figure 2b).

The polyIn‐BTB crystallizes in the monoclinic *P*2_1_/n space group with its asymmetric unit consisting of four BTB^3−^ ligands (named BTB‐1, BTB‐2, BTB‐3, BTB‐4, respectively), four and a half In^3+^ ions, four and a half [NH_2_(CH_3_)_2_]^+^ ions, two coordinated water molecules, two coordinated DMSO molecule and three bridging oxygen atoms (Figure S4, Supporting Information). Different from monoIn‐BTB, polyIn‐BTB includes three different connect mode metal clusters (cluster 1, cluster 2, and cluster 3). Two In1 and one In2 are connected by four bridging carboxylates and two µ2‐O atoms to form a linear cluster [In_3_O_2_(COO)_4_] (cluster 1), two In3 and two In4 are connected by four carboxylates and two µ‐O atoms to form a planar cluster [In_3_O_2_(COO)_4_] (cluster 2), two In5 and two In6 connected by six carboxylates and two µ‐O atoms to from a corrugated cluster [In_3_O_2_(COO)_6_] (cluster 3) (Figure 2c; Figure S5, Supporting Information). Each BTB linker is connected to one type of metal cluster with a monodentate, bridging, or chelating pattern, and each metal cluster connects two of each BTB to form a 3D super octahedron cage network (Figures S6–S8, Supporting Information). The 3D network generates pores with diameters of 23.3 Å, capable of housing an additional layer of the 3D network, thereby forming a two‐layer intercalated structure (Figure 2d). The adjacent BTB layers are separated by a distance of 3.7 Å, suggesting a *π*–*π* interaction between them (Figure S9, Supporting Information).

To further determine the crystal structure of monoIn‐BTB and polyIn‐BTB, X‐ray diffraction (XRD), Fourier transform infrared (FT‐IR), scanning electron microscopy (SEM), Brunauer–Emmett–Teller (BET) and Zeta potential were performed. The simulated XRD and experimental XRD patterns of monoIn‐BTB and polyIn‐BTB are shown in Figure 2e. The experimental XRD patterns correspond well with the simulated results from the single crystal data, indicating the phase purity of the synthesized samples. FT‐IR spectrum of H_3_BTB, monoIn‐BTB, and polyIn‐BTB are shown in Figure 2f. The weak stretching vibration peak of H_3_BTB at 1711 cm^−1^ suggests that the protonated H_3_BTB ligand reacts with In^3+^.^[^ ^36^, ^37^ ^]^ The stretching vibration peak at 1576 cm^−1^ is characteristic of asymmetric C═O stretching vibrations,^[^ ^38^ ^]^ indicating the presence of a carboxyl functional group, which can provide the coordination active site. SEM reveals that monoIn‐BTB exhibits a triangular prismatic rod morphology (Figure 2g), and polyIn‐BTB displays a willow, diamond‐like morphology (Figure 2h). The BET surface area of polyIn‐BTB is 948.88 m^2^g^−1^, significantly higher than that of monoIn‐BTB (11.22 m^2^g^−1^) (Figure S10, Supporting Information), which is beneficial to expose more metal active sites.^[^ ^39^ ^]^ The Zeta potential of polyIn‐BTB is 8.49 mV, which is beneficial for the adsorption of Escherichia Coli (−20–−40 mV) and Methicillin‐Resistant Staphylococcus aureus (zeta potential is −30 to −50 mV) (Figure S11, Supporting Information).^[^ ^40^ ^]^ Then, the produced ROS over the surface of polyIn‐BTB can quickly kill bacteria.^[^ ^41^ ^]^

### 2.2 DFT Theoretical Calculation of monoIn‐BTB and polyIn‐BTB

**Figure 3:**
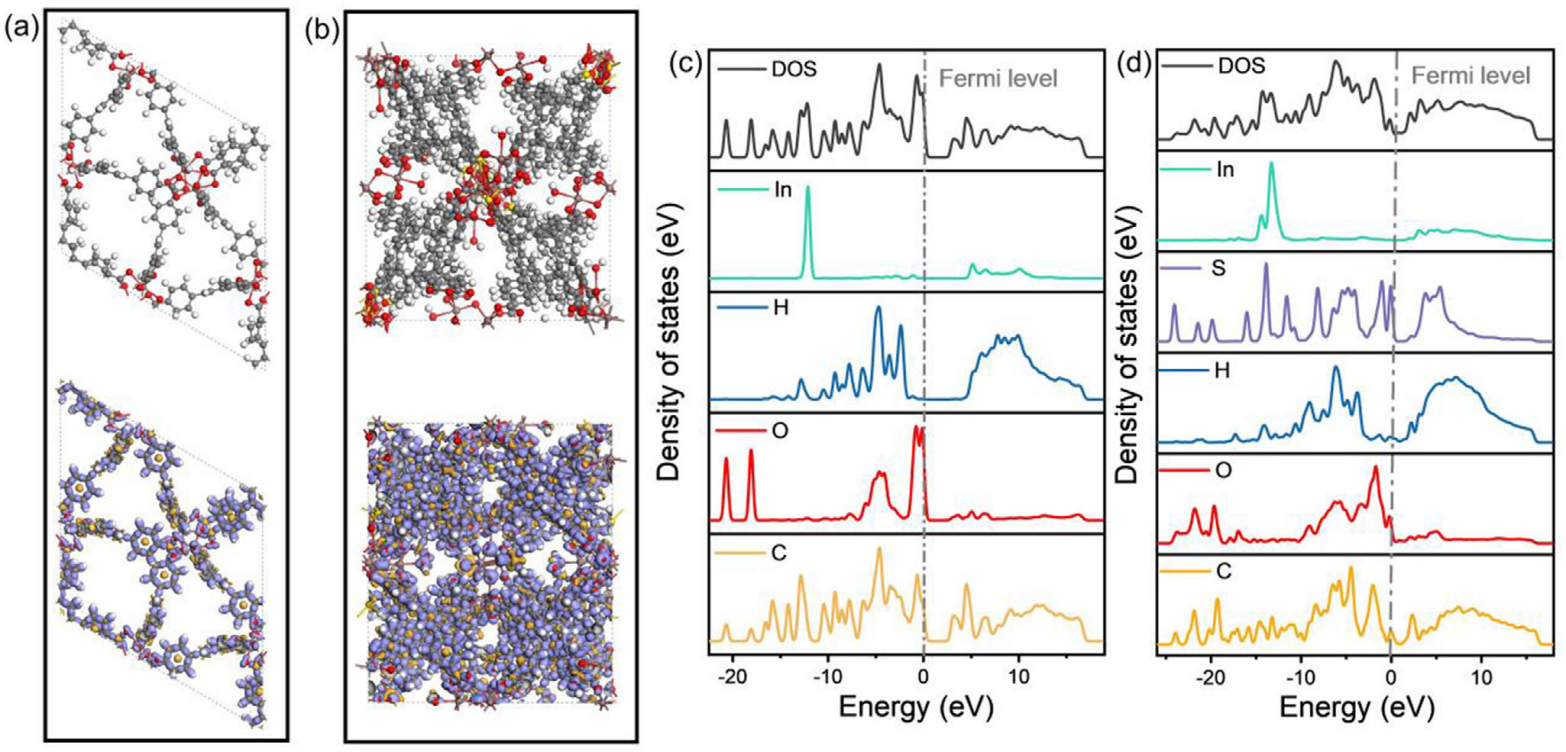
a,b) The electron differential density map of (a) monoIn‐BTB and (b) polyIn‐BTB. c,d) PDOS of all atoms for (c) monoIn‐BTB and (d) polyIn‐BTB.

To reveal the electronic structure of polyIn‐BTB and monoIn‐BTB, DFT was carried out to study the electron distribution of polyIn‐BTB and monoIn‐BTB. The electronic difference density map of polyIn‐BTB shows that the electronic distribution has been rearranged in the polymetallic nodes, confirming that the construction of multi‐nuclear clusters enhances the electron density of the metal site (**Figure** **3**a,b), which is conducive to the adsorption of O_2_ and H_2_O over MOFs for the production of ROS. In contrast, the electron difference density is relatively uniform in monoIn‐BTB, indicating weaker electronic localization. Further, the Mulliken charge distribution reveals that multi‐nuclear metal clusters significantly reduce the electron density of oxygen atoms, which is favorable for the generation of ROS (Figures S12 and S13, Supporting Information). The d‐band center of polyIn‐BTB is −2.23 eV, far from the Fermi level, compared to monoIn‐BTB (d‐band center of −0.81 eV), which is beneficial for facilitating the effective desorption of ROS for enhancing antibacterial activity^[^ ^42^, ^43^, ^44^ ^]^ (Figure 3c,d).

### 2.3 In Vitro Antibacterial Activity of monoIn‐BTB and polyIn‐BTB

**Figure 4:**
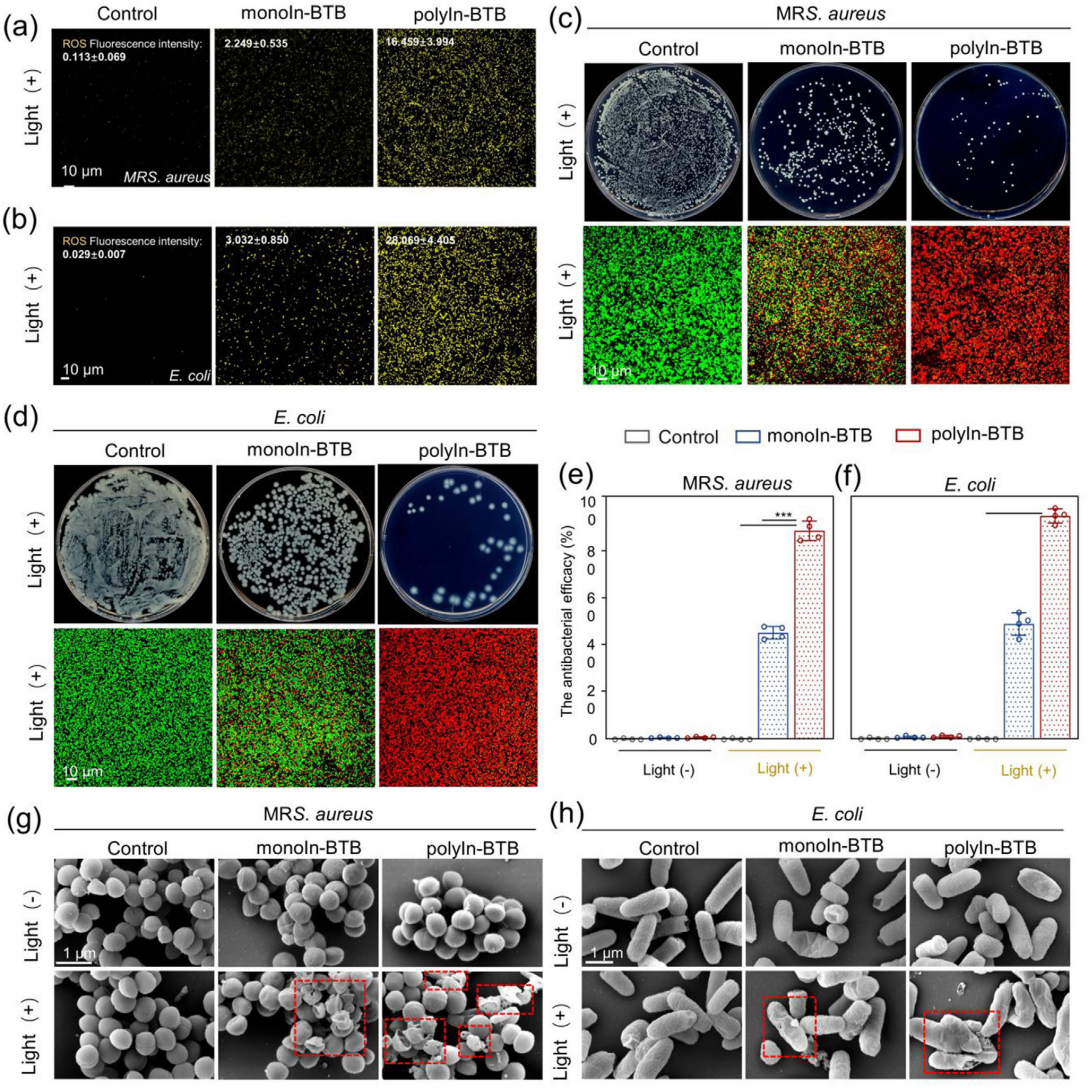
a–d,g–h) ROS fluorescent images, bacterial colonies, live/dead bacterial staining, and SEM images of *MRS. aureus* (a,c,g) and *E. coli* (b,d,h) after different treatments. (c–f) The quantitative antibacterial efficacy in (c,d) was shown in (e,f). (Dotted box refers to the rupture of the bacterial cell membrane, ^***^ *p* < 0.001).

To evaluate the antibacterial activity of polyIn‐BTB and monoIn‐BTB, the total ROS yield of polyIn‐BTB and monoIn‐BTB in Methicillin‐Resistant *Staphylococcus aureus* (*MRS. aureus*) and *Escherichia Coli* (*E. coli*) was measured. Under the dark condition, negligible ROS is generated over polyIn‐BTB and monoIn‐BTB, indicating weak antibacterial activity (Figures S14 and S15, Supporting Information). However, the concentration of ROS over polyIn‐BTB is significantly increased relative to the control and monoIn‐BTB group after 30 min of light irradiation, which can promote antibacterial activity (**Figure** **4**a,b). Meanwhile, the polyIn‐BTB also exhibits a high ROS formation concentration relative to monoIn‐BTB, which is beneficial for enhancing the antibacterial activity (Figure S16, Supporting Information). The antibacterial ability of all nanoenzymes was assessed by using the spread plate method. Under dark conditions, all groups exhibit a negligible reduction in bacterial colonies across, implying minimal antibacterial activity without light (Figures S17 and S18, Supporting Information). In contrast, after 30 min of light irradiation, polyIn‐BTB displays a superior antibacterial efficacy against both *MRS. aureus* and *E. coli* relative to monoIn‐BTB (Figure 4c,d). Consistently, live/dead bacterial staining reveals the lowest proportion of live bacteria (green fluorescence) in the polyIn‐BTB group under light irradiation, demonstrating their more efficient antibacterial efficacy compared to monoInBTB (Figure 4c,d). Figure 4e,f shows the quantitative antibacterial efficacy of all samples. Under light radiation conditions, the antibacterial efficiency of polyIn‐BTB against *MRS. aureus* and *E. coli* can reach 87.0% and 92.0%, respectively, which is significantly higher than that of monoIn‐BTB (42% of *MRS. aureus* and 50% of *E. coli*, respectively) and most reported literatures (Figure S19, Supporting Information). In order to demonstrate their ROS‐dependent antibacterial effects, the ROS inhibitors (Superoxide dismutase and Catalase) were added into the antibacterial system. We found that the antibacterial efficacy of both polyIn‐BTB and monoIn‐BTB under light irradiation is significantly diminished (Figure S20, Supporting Information), suggesting the key role of ROS on the antibacterial. Further, XRD and FTIR results confirm the excellent crystal structure and chemical bond stability of polyIn‐BTB after the antibacterial tests (Figure S21, Supporting Information). The time‐dependent inductively coupled plasma mass spectrometry (ICP‐MS) results confirm that no In^3+^ is dissolved into the culture medium during the antibacterial process, excluding potential bactericidal contributions from ionic dissolution (Figure S22, Supporting Information). The electron paramagnetic resonance (EPR) results indicate that hydroxyl radicals (^•^OH) and singlet oxygen (^1^O_2_) are the main ROS antibacterial species (Figure S23, Supporting Information). Moreover, SEM was used to reveal the antibacterial mechanism of antibacterial mechanism, showing that the *MRS. aureus* and *E. coli* in the polyIn‐BTB group exhibit disrupted membrane structures and signs of rupture relative to the monoIn‐BTB group (Figure 4g,h), confirming that the antibacterial mechanism is based on the ROS disrupting the cell membrane structure.

### 2.4 Antibacterial Activity Assessment in the Wound Healing Model

**Figure 5:**
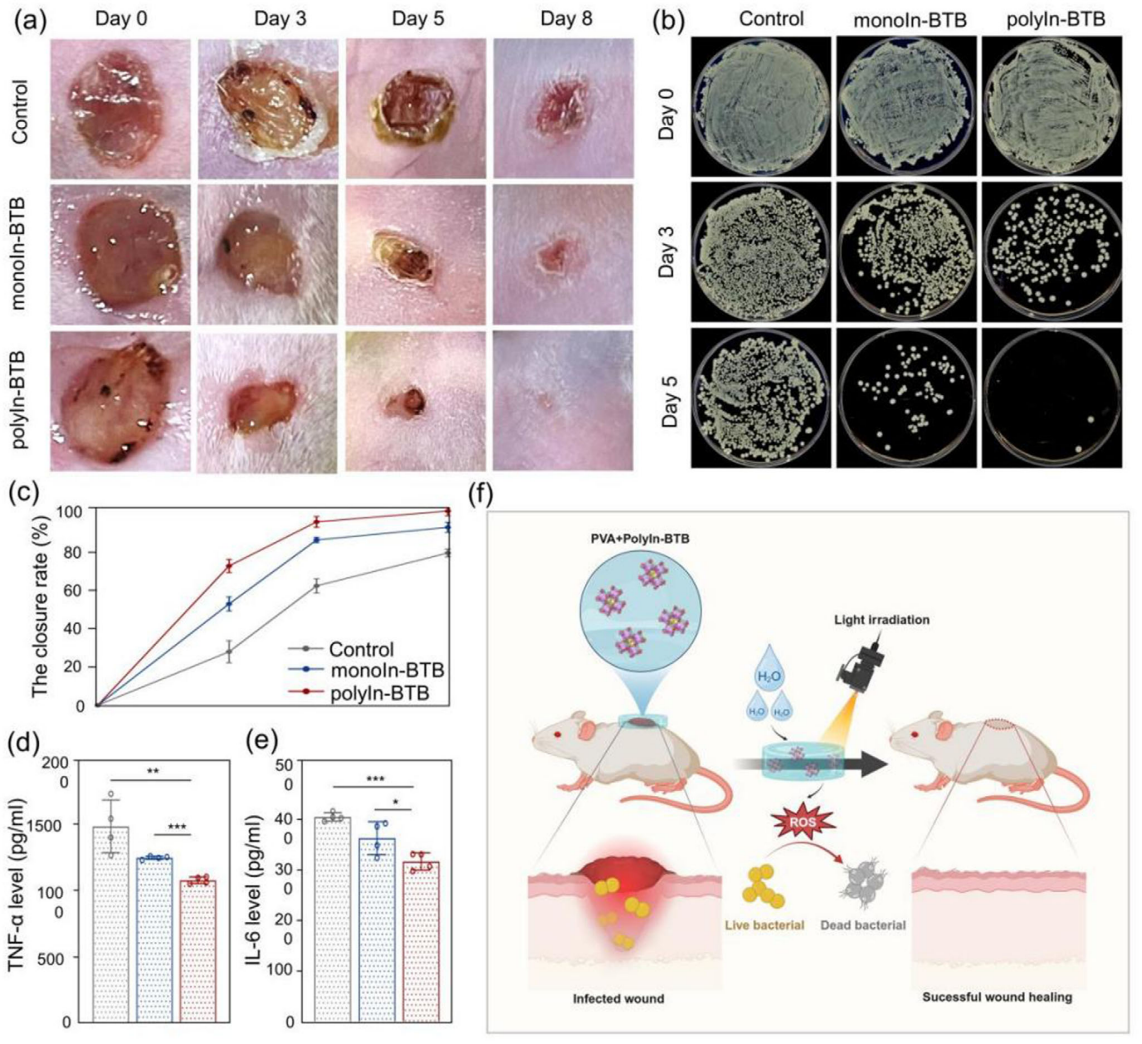
a) Photographs of the infected skin wounds treated with polyIn‐BTB and monoIn‐BTB. b) Representative images of *MRS. aureus* colonies in the different groups. c) Quantification of the wound healing rate in all groups. d,e) The inflammatory cytokines TNF‐α and IL‐6 levels on day 3 after different treatments. f) Schematic illustration of skin wound infection model treated by polyIn‐BTB. (^*^ *p* < 0.05, ^**^ *p* < 0.01, ^***^ *p* < 0.001).

To further assess the antibacterial activity of polyIn‐BTB, the infected wounds in vivo as the experimental model was chosen. **Figure** **5**a shows the photographs of the infected skin wounds treated with polyIn‐BTB and mono‐InBTB. In the polyIn‐BTB group, the wound healing rate is ≈98.0% closure on 8 days, significantly better than the monoIn‐BTB group (79.0%) (Figure 5b). To assess the antibacterial efficacy of polyIn‐BTB in vivo, the bacterial burden around the wounds was quantified on days 0, 3, and 8 by enumerating bacterial colonies. The bacterial colony count in the polyIn‐BTB group is significantly lower than that in the monoIn‐BTB and control groups (Figure 5c). Moreover, the inflammatory cytokines TNF‐α and IL‐6 levels are notably reduced in the polyIn‐BTB group on day 3 as compared to the other two groups, indicating reduced inflammation in the early stage of healing (Figure 5d,e). Hematoxylin‐eosin (HE) staining results on day 8 reveal that the infected skin treated with the polyIn‐BTB exhibits healing closely to the healthy tissue, showing a moderate epidermal thickness, well‐defined epidermal ridges, and the re‐establishment of skin appendages (Figure S24, Supporting Information). Consistent with the in vitro results, the in vivo results demonstrate that polyIn‐BTB exhibits remarkable antibacterial activity and efficient wound healing relative to monoIn‐BTB, confirming that the construction of multi‐nuclear metal coordination nodes promotes antibacterial and wound healing. The above results highlight the excellent therapeutic potential of polyIn‐BTB in treating infected wounds under visible light irradiation (Figure 5f), which confirms our new strategy of polymetallic coordination nodes is promising for developing nanoenzymes with high performance.

## 3 Conclusion

In summary, to enhance the antibacterial of drug‐resistant bacteria over MOFs, we proposed a new strategy of constructing multi‐nuclear metal coordination nodes for enhancing photocatalytic ROS production. DFT theoretical calculation shows that the as‐made polyIn‐BTB exhibits higher electron density compared to monoIn‐BTB, which is beneficial for facilitating ROS generation. In vitro experiments confirm that more ROS production over polyIn‐BTB, relative to monoIn‐BTB, promotes the antibacterial of drug‐resistant bacteria. The as‐made polyIn‐BTB exhibits antibacterial efficiencies of 87.0% and 92.0% for *MRS. Aureus* and *E. coli*, respectively, which is significantly higher than that of monoIn‐BTB, confirming that the construction of multi‐nuclear metal coordination nodes boosts the antibacterial performance. Furthermore, by applying polyIn‐BTB to wound treatment, the wound healing rate can reach 98.0% in 8 days, which is also better than monoIn‐BTB. Overall, this work brings a new strategy of constructing multi‐nuclear metal coordination nodes for enhancing the antibacterial of drug‐resistant bacteria, which has guiding significance for rationally designing the nanozyme with high performance.

## Conflict of Interest

The authors declare no conflict of interest.

## Supporting information



Supporting Information

## Data Availability

The data that support the findings of this study are available from the corresponding author upon reasonable request.
