# Supplementary material for: Constructing Polymetallic Nodes in Metal–Organic Frameworks Enhance Antibacterial of Drug‐Resistant Bacteria
Source: Adv Sci (Weinh). 2025 Apr 26;12(25):2501327. doi: 10.1002/advs.202501327 (PMC12224937; doi:10.1002/advs.202501327)
Supplement: Supplementary file 1 — Supporting Information [file ADVS-12-2501327-s001.pdf]

ADVANCED SCIENCE
Open Access

Supporting Information

for *Adv. Sci.*, DOI 10.1002/advs.202501327

Constructing Polymetallic Nodes in Metal–Organic Frameworks Enhance Antibacterial of Drug-Resistant Bacteria

Qinqin Li, Shihan Zhang, Yachao Xu, Yaru Guo and Youxing Liu**

Supporting Information

Constructing Polymetallic Nodes in Metal-Organic Frameworks Enhance Antibacterial of Drug-Resistant Bacteria

Qinqin Li[1#], Shihan Zhang[2#], Yachao Xu[3], Yaru Guo[2]*, Youxing Liu[1]*

[1]Beijing Key Laboratory of Electrochemical Process and Technology for Materials, Beijing University of Chemical Technology, Beijing 100029, P. R. China

[2]Department of Geriatric Dentistry, Peking University School and Hospital of Stomatology, Beijing 100081, P. R. China

[3]School of Materials Science and Engineering, Peking University, Beijing 100871, China
#These authors contributed equally
*E-mail Author: kqguoyaru@bjmu.edu.cn; liuyxpost@pku.edu.cn

Materials and methods

Materials and Instrumentation

1,3,5-tri(4-carboxyphenyl)benzene (H_3BTB, 98%), $In(NO_3)_3·4.5H_2O$ (99%), dimethyl sulfoxide (DMSO, 99.9%), *N*, *N*-dimethylformamide (DMF, 99.9%), *N*, *N*-dimethylacetamide (DMA, 99.9%), nitric acid (96%) were purchased from Energy Chemical Co. Ltd, Beijing Innochem Technology Co., Ltd, and Shanghai Bide Pharmaceutical Technology Co., Ltd.. All commercial reagents were used as received without further purification.

Elemental analyses were performed with a Perkin-Elmer 240C elemental analyzer. Fourier Transform infrared spectroscopy (FT-IR) spectra were recorded from KBr pellets in the range of 4000-400 cm^{-1} on a Bruker Vector 22 spectrometer. Thermogravimetric analyses (TGA) were carried out on a Mettler-Toledo thermal analyzer with a heating rate of 10 °C/min in the N_2 atmosphere. Powder X-ray diffraction patterns (PXRD) were performed on a Smart Lab X-ray diffractometer with Cu Kα radiation (λ = 1.5418 Å), in which the X-ray tube was operated at 40 kV and 40 mA. Scanning electron microscopy (SEM) was obtained on FlexSem 1000. The electron paramagnetic resonance (EPR) was obtained on Bruker A300-10.

Synthesis of $(NH_2Me_2)_3[In_3(BTB)_4]·12DMA·4.5H_2O$ (monoIn-BTB)

A mixture of H_3BTB (17.5 mg, 0.04 mmol) and $In(NO_3)_3·4.5H_2O$ (7.6 mg, 0.02 mmol) in DMA (1.0 mL) and HNO_3 (0.015 mL) were sealed in a 23mL Parr Teflon-lined stainless steel vessel, heated at 125 °C for 36 h, and then slowly cooled to room temperature; Rod-like yellow crystals of monoIn-BTB were obtained in 49% yield (based on $In(NO_3)_3·4.5H_2O$). Anal. Calcd for MonoIn-BTB: C, 58.07; H, 6.05; N, 6.27%; Found: C, 57.91; H, 6.15; N, 6.21%.

Synthesis of $(NH_2Me_2)_9[In_9O_6(BTB)_8(H_2O)_4(DMSO)_4]·27DMSO·21H_2O$ (polyIn-BTB)

A mixture of H_3BTB (17.5 mg, 0.04 mmol) and $In(NO_3)_3·4.5H_2O$ (7.6 mg, 0.02 mmol) in DMSO (0.25 mL), DMF (0.15 mL), H_2O (0.025 mL) and HNO_3 (0.017 mL, 1M in DMSO and DMF) were sealed in a 23mL Parr Teflon-lined stainless steel vessel, heated at 125 °C for 36 h, and then slowly cooled to room temperature; Diamond-like colorless crystals of polyIn-BTB were obtained in 61% yield (based on $In(NO_3)_3·4.5H_2O$). Anal. Calcd for MonoIn-BTB: C, 45.00; H, 5.46; N, 1.59; S, 12.58%; Found: Found: C, 44.79; H, 5.63; N, 1.42; S, 13.07%.

Density functional theory (DFT) calculation details

The DFT calculations have been carried out with the projector augmented wave (PAW) method using the Perdew-Burke-Ernzerhof (PBE) exchange-correlation functional for the geometry optimizations and self-consistent total energy calculations. To describe the on-site Coulomb interaction among the localized zinc 3delectrons, we adopted the GGA approximation with an effective Hubbard U-parameter (Ueff = 8.5 eV). Calculations were performed with the

Cambridge Sequential Total Energy Package. The plane wave cut-off was set at 400 eV, and the total energy convergence at 10^{-6} eV for the self-consistent iterations. The Brillouin zone is sampled by a Γ-centered k-mesh of 2×1×2 and the convergence is tested by varying the mesh size. Structures were fully relaxed until the maximum force on each atom becomes less than 0.02 eV/Å[1].

The d-band center is calculated using the following equation. Experimentally, the surface valence band photoemission spectra are corrected by subtracting the Shirley background[2]. The upper level of integration for background subtraction was consistently set at the 10.0 eV binding energy position across all valence-band spectra to ensure the accurate comparison of all valence-band spectra. Theoretical calculations involve evaluating the d bands of MOF-based catalysts based on the centroid of the densities of states projected onto the 3d orbitals of the metal atoms using a similar equation.

$$\mu = \frac{\int E \cdot D(E)dE}{\int D(E)dE} - E_{Fermi}$$

Where E is the electron energy, D (E) is the DOS projected onto the 3d orbitals of metal atoms, and the E_{Fermi} is the Fermi energy.

Antibacterial Activity of monoIn-BTB and polyIn-BTB *In Vitro*

Methicillin-resistant Staphylococcus aureus (*MRS. aureus*) and the Gram-negative bacterium Escherichia coli (*E. coli*) were selected in this experiment. One group of samples (Control, polyIn-BTB, and monoIn-BTB) was treated without light irradiation, while another group was exposed to Xenon lamp (300Mw cm^{-2}, Microsolar 300, Beijing Perfectlight). The concentration of polyIn-BTB and monoIn-BTB used for the antimicrobial test was 500 μg mL^{-1}. After 30 minutes of irradiation, the antimicrobial activity of polyIn-BTB and monoIn-BTB against *MRS. aureus* and *E. coli* were assessed using the plate count method, live/dead bacterial staining assay (Thermo Fisher, USA), and scanning electron microscopy (SEM). The antibacterial efficacy was quantified using Image J software.

Reactive oxygen species (ROS) detection

To detect the production of ROS under the Xenon lamp, 2′,7′-dichlorofluorescein diacetate (MedChemExpress, USA) was used to react with ROS and generate fluorescence. The reaction and measurement procedures were carried out according to the manufacturer's protocol. Fluorescence images were acquired using a confocal microscope at 63x magnification (STELLARIS-8 and TCS SP8, Leica). At least three randomly selected fields were analyzed for each group using ImageJ.

***In vivo* animal wound healing tests**

All animal surgical procedures were approved by the Institutional Animal Care and Use Committee of Peking University (DLASBD0252). Male BALB/c mice (4–6 weeks old) were purchased from Vital River Laboratories (Beijing, China). Prior to the experiment, all mice were housed under appropriate conditions and divided into three groups: Control, polyIn-BTB and monoIn-BTB. Two time points (day 3 and 8) were set, with at least three mice in each group. After anesthesia with 1% sodium pentobarbital solution, a full-thickness skin wound (6 mm in diameter) was created on the back of each mouse. After that, 100 μL of an S. aureus solution (1×10^6 CFU) was applied to the wound to establish the infected wound model. One day later, hydrogels composed of 300 mg mL^{-1} polyvinyl alcohol (PVA) mixed with polyIn-BTB (5 μg/mL) or monoIn-BTB (5 μg mL^{-1}) were applied to the wounds and irradiated with Xenon lamp for 30 minutes. The wounds in the control group were treated with pure PVA. The application of the materials was designated as day 0. On days 0, 3, 5, and 8, photographs of the wounds were taken, and local tissue fluid was collected from each group for the plate count test. The animals were euthanized by CO_2 inhalation on day 3 to evaluate the expression of inflammatory cytokines using TGF-α and IL-6 Elisa kits (Qisong, China) according to the manufacturer's protocol. The wound closure rate was analyzed using Image J software. Schematic illustrations were created using BioRender.com.

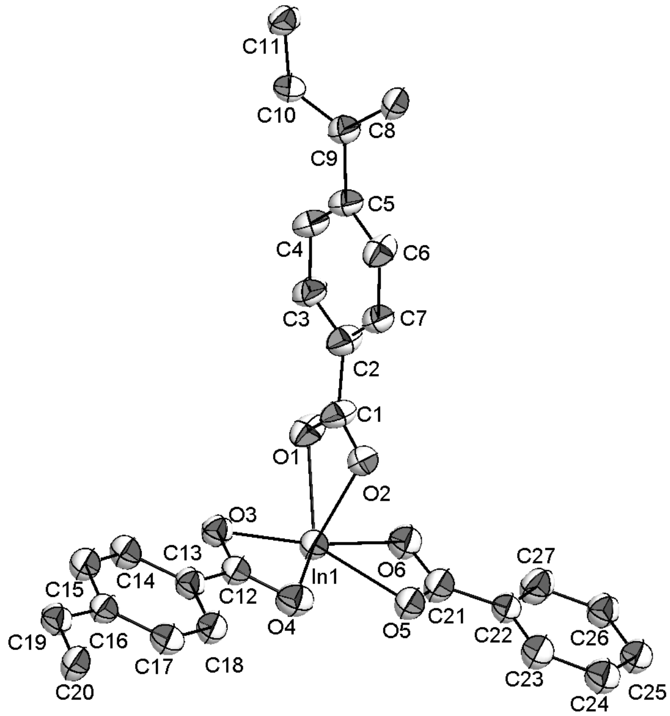

Figure S1. The asymmetric unit of monoIn-MOF with 50% probability (Hydrogen atoms are omitted for clarity).

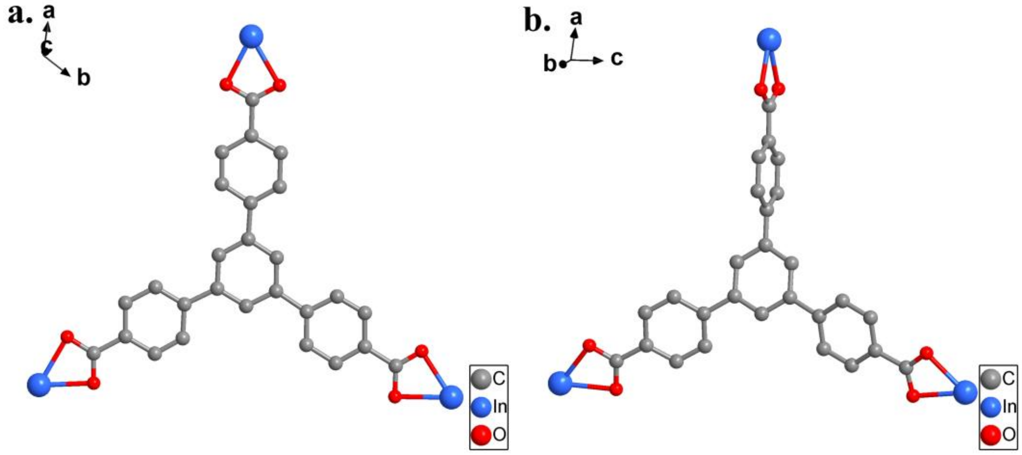

Figure S2. (a and b) The coordination environment of (a) BTB-1 and (b) BTB-2 ligands in monoIn-MOF (Hydrogen atoms are omitted for clarity).

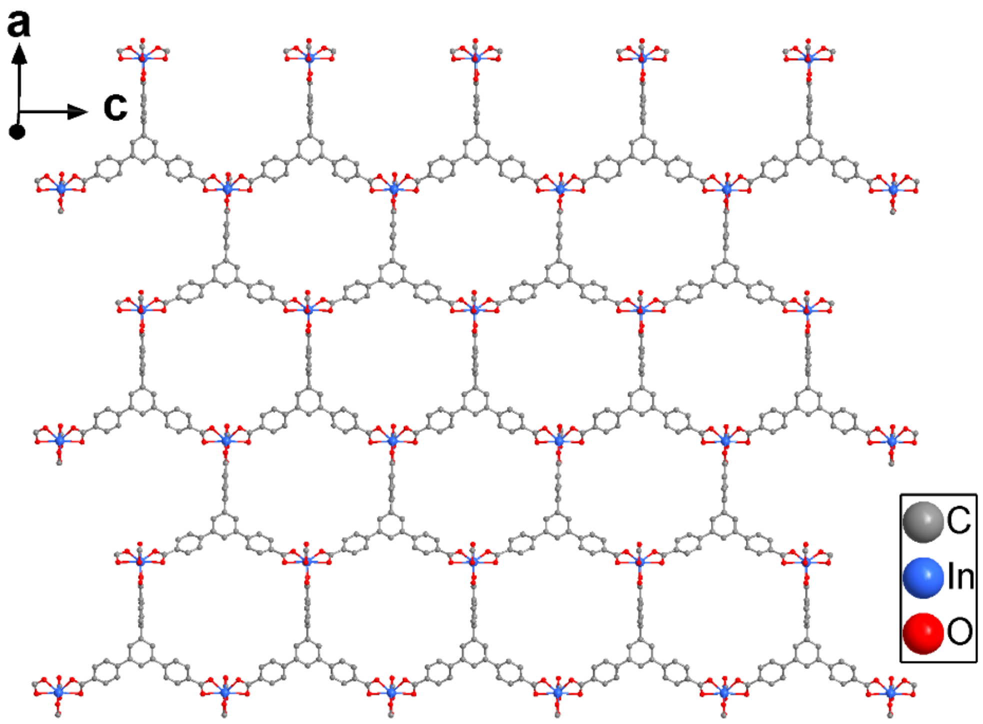

Figure S3. The single 3D network structure of monoIn-BTB along the *b*-axis (Hydrogen atoms are omitted for clarity).

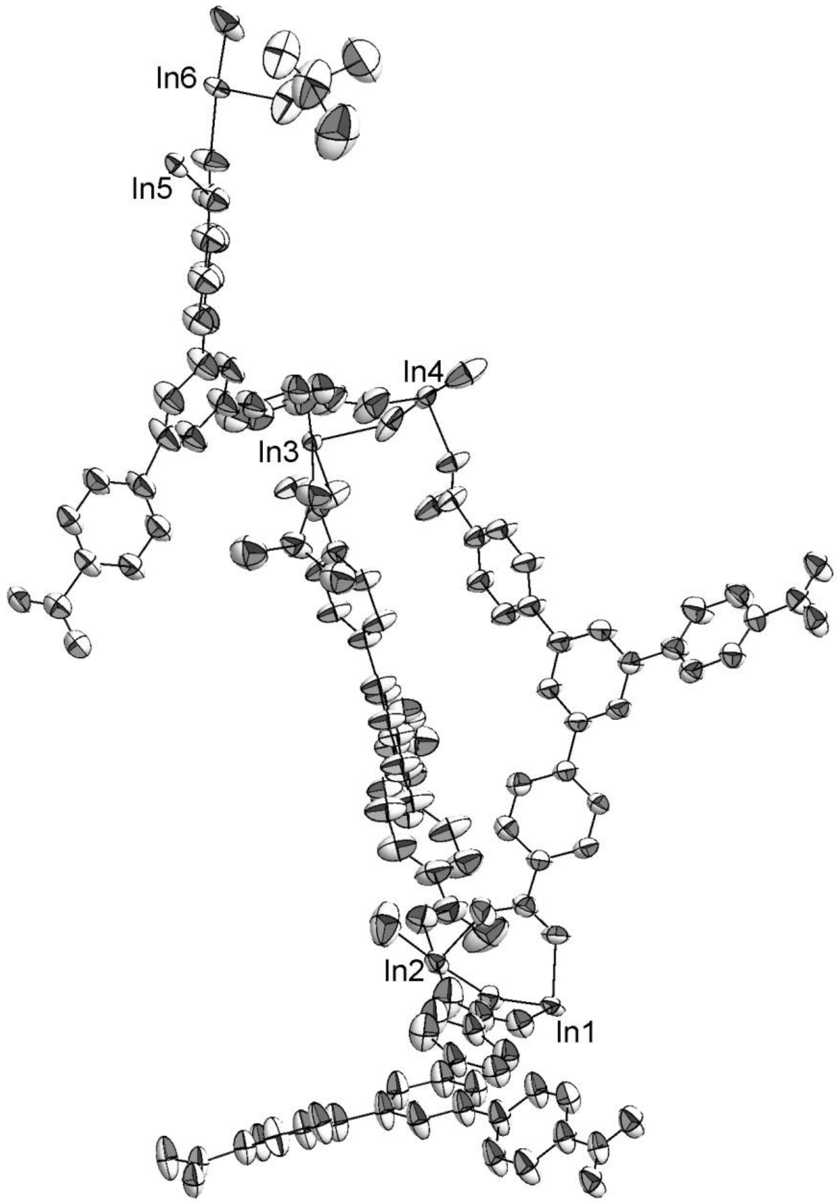

Figure S4. The asymmetric unit of polyIn-MOF with 50% probability (Hydrogen atoms are omitted for clarity).

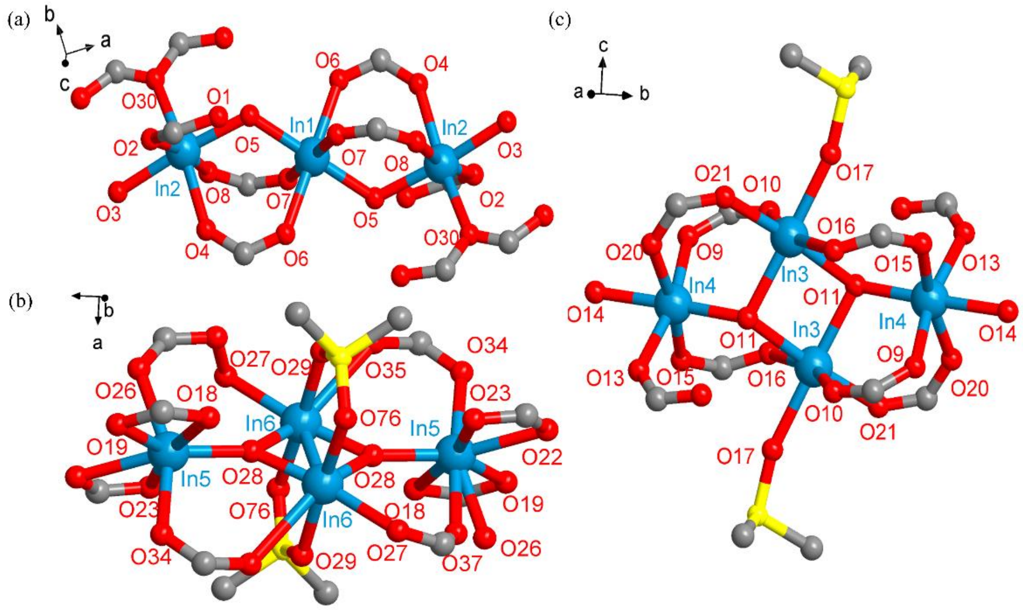

Figure S5. (a-c) The coordination environment of (a) metal clusters 1, (b) metal cluster 2 and (c) metal cluster 3 in polyIn-BTB.

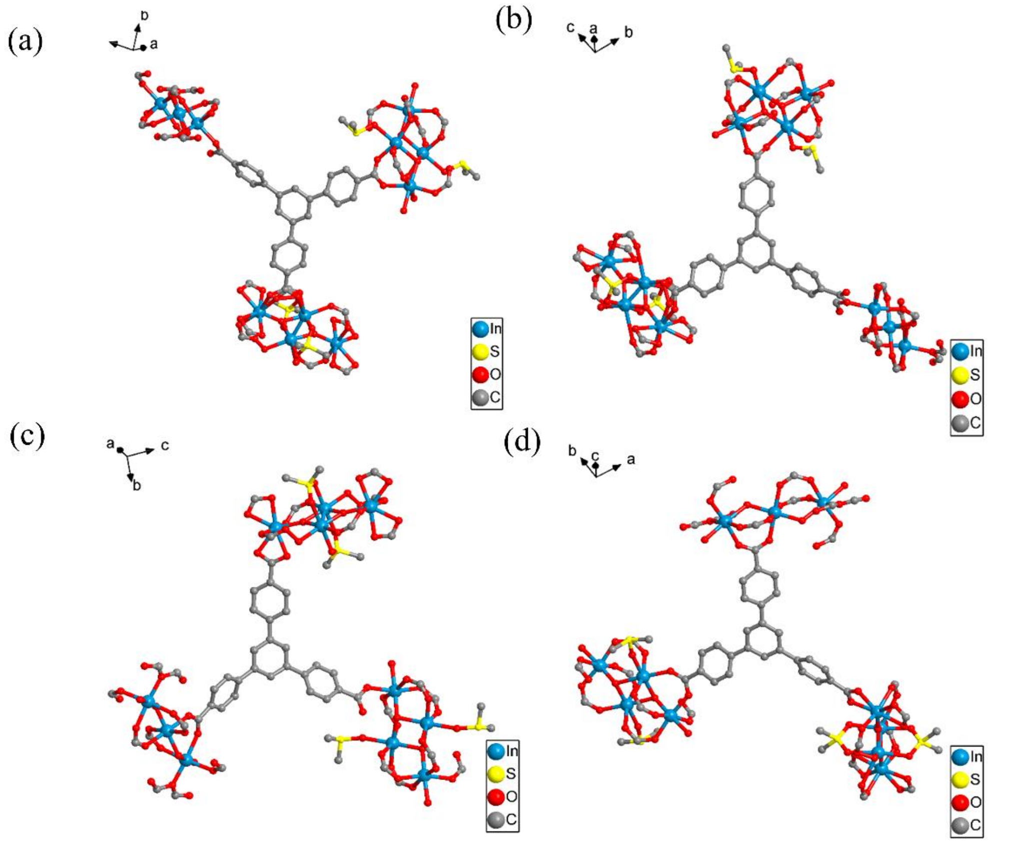

Figure S6. (a-d) The coordination environment of (a) BTB-1, (b) BTB-2, (c) BTB-3 and (d) BTB-4 in polyIn-BTB.

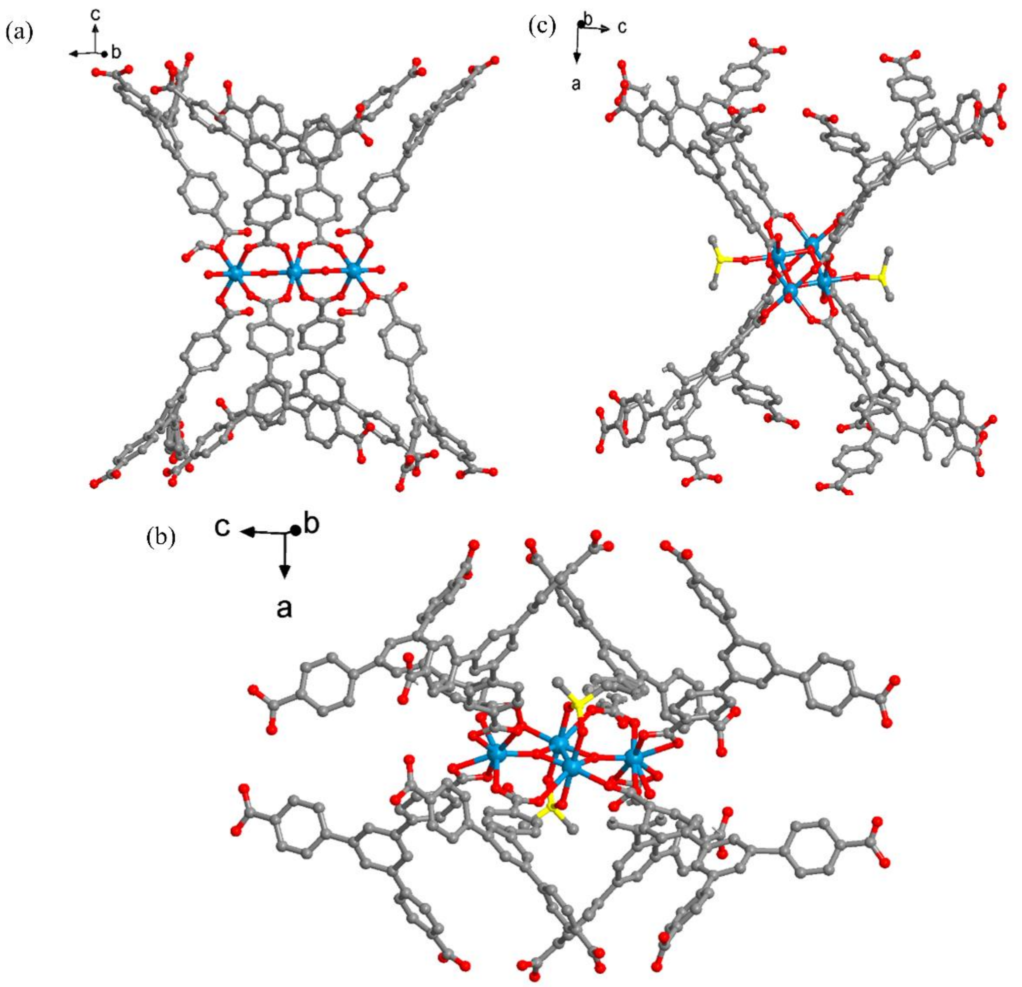

Figure S7. (a-c) The coordination mode of (a) metal clusters 1, (b) metal cluster 2 and (c) metal cluster 3 with BTB in polyIn-BTB.

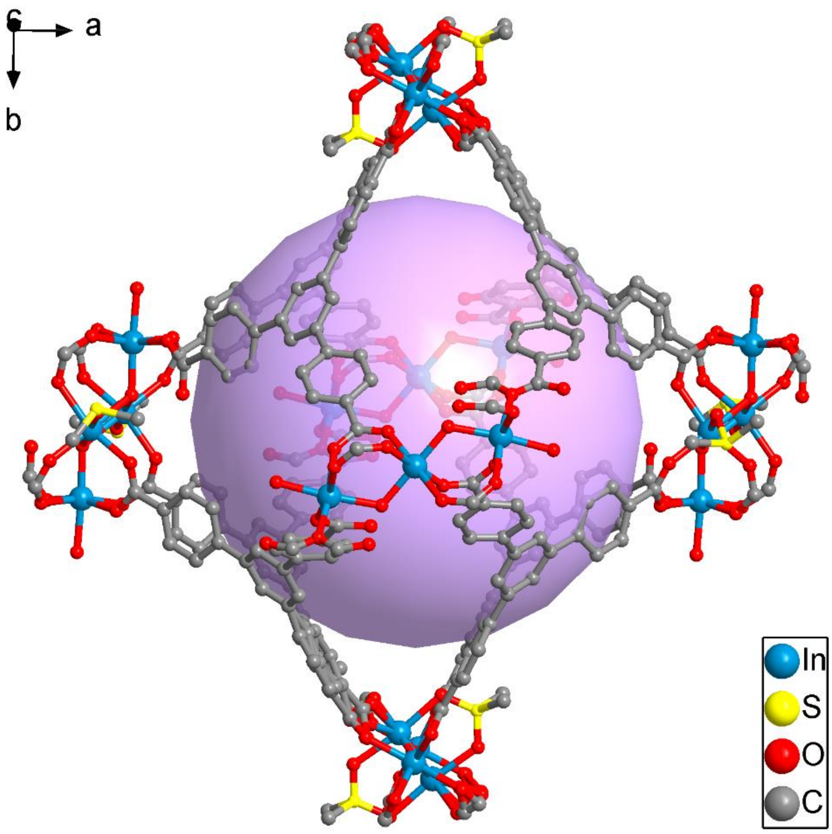

Figure S8. The octahedral cage in polyIn-BTB.

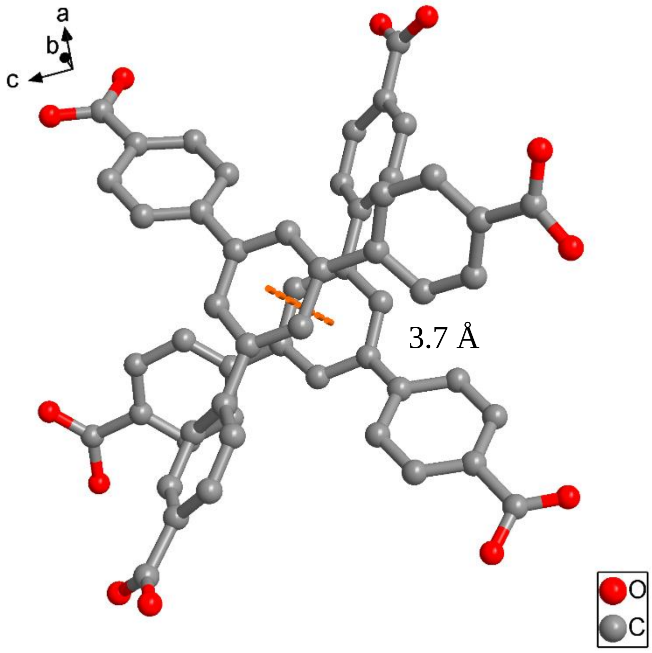

Figure S9. The π-π interaction between adjacent BTB of polyIn-BTB.

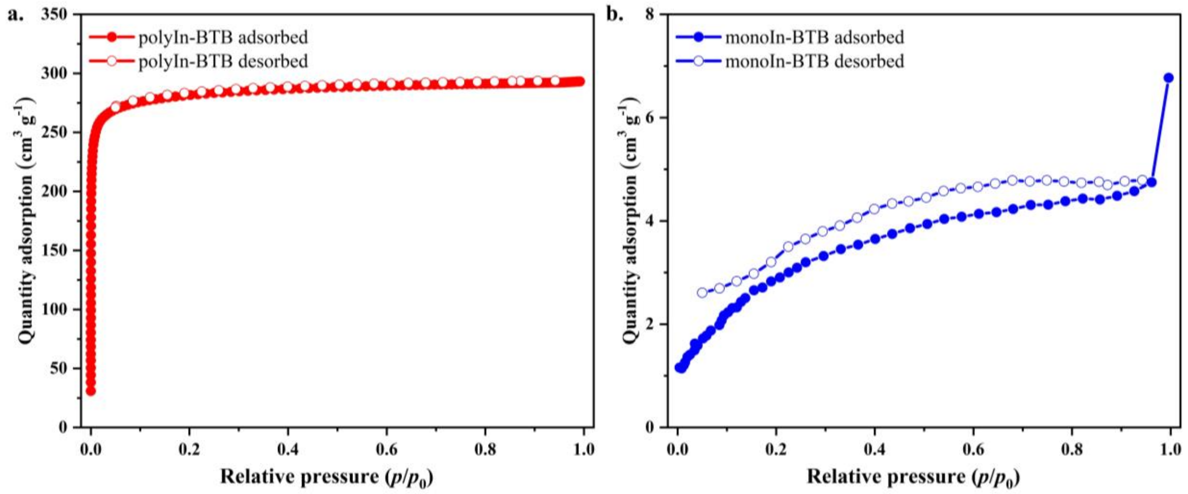

Figure S10. N_2 adsorption–desorption isotherms of (a) polyIn-BTB and (b) monoIn-BTB.

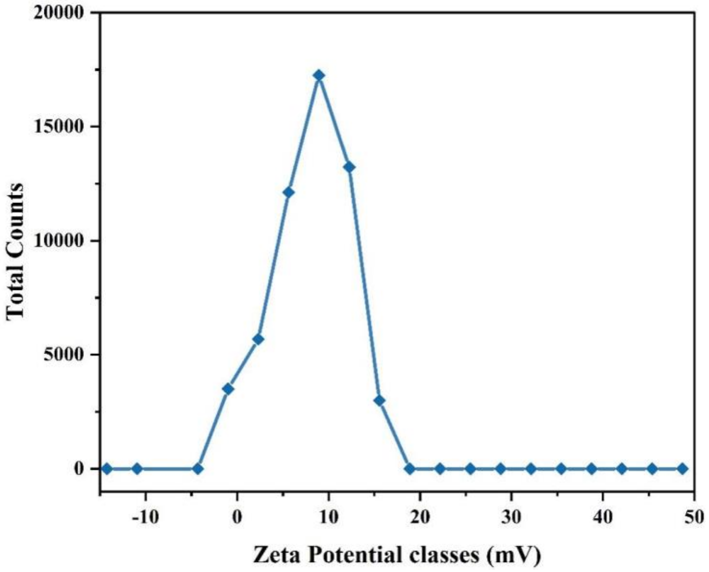

Figure S11. The zeta potential of polyIn-BTB.

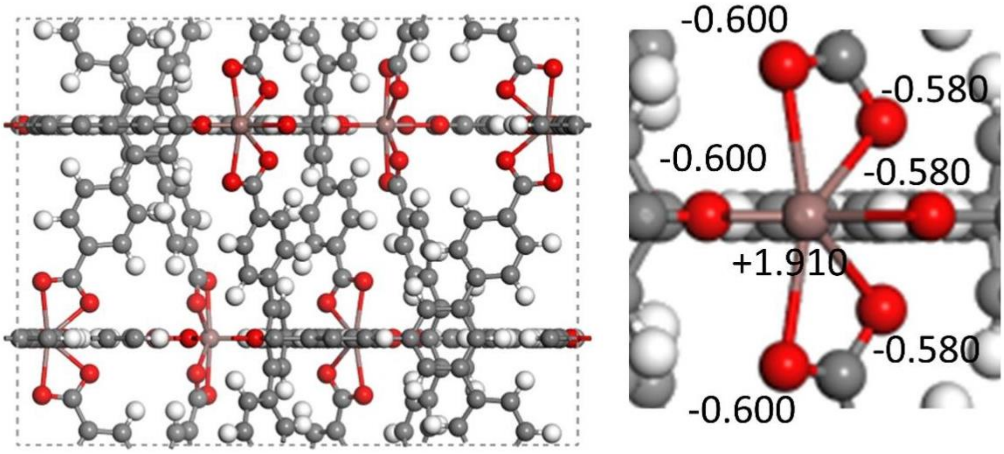

Figure S12. The Mulliken charge distribution of monoIn-BTB (unit: eV).

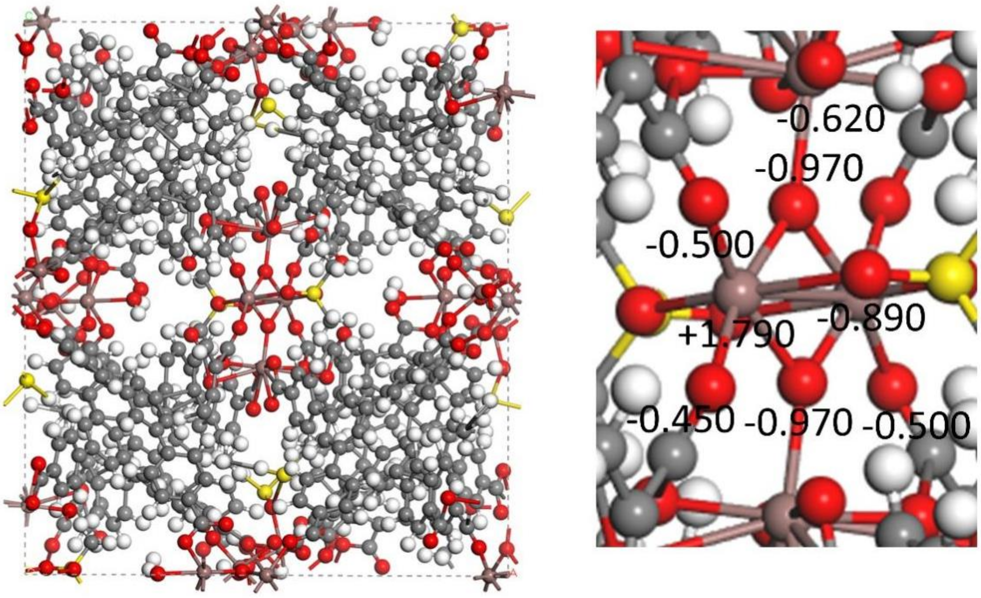

Figure S13. The Mulliken charge distribution of polyIn-BTB (unit: eV).

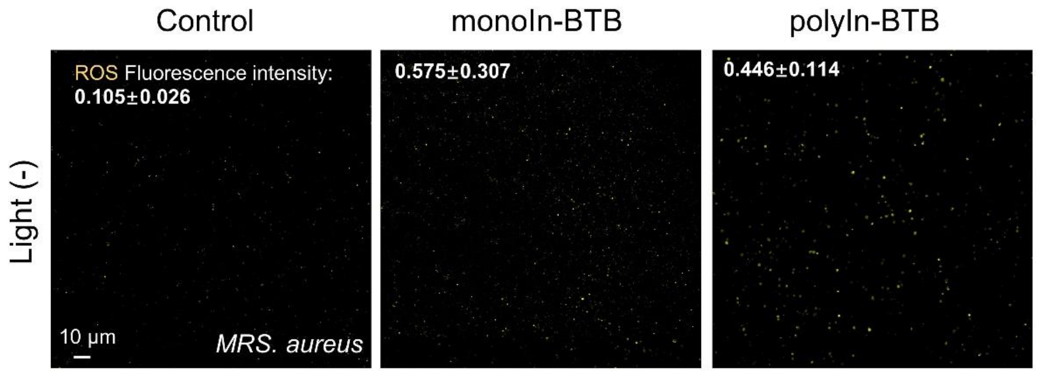

Figure S14. ROS fluorescent images of *MRS. aureus* after different treatments.

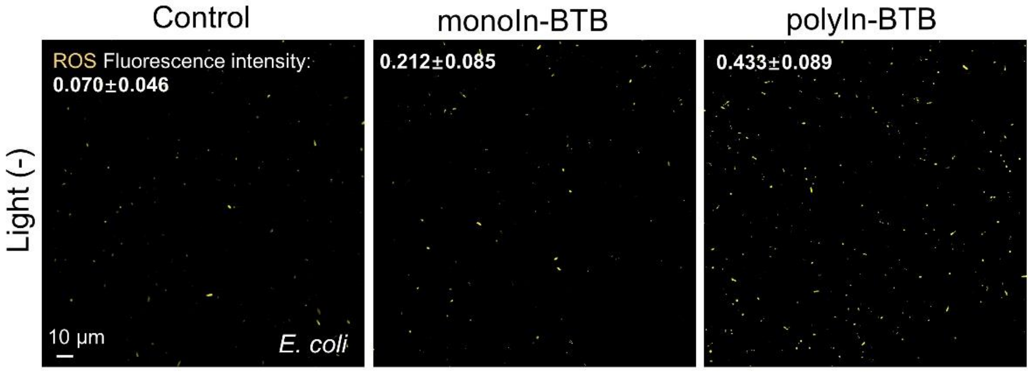

Figure S15. ROS fluorescent images of *E. coli* after different treatments.

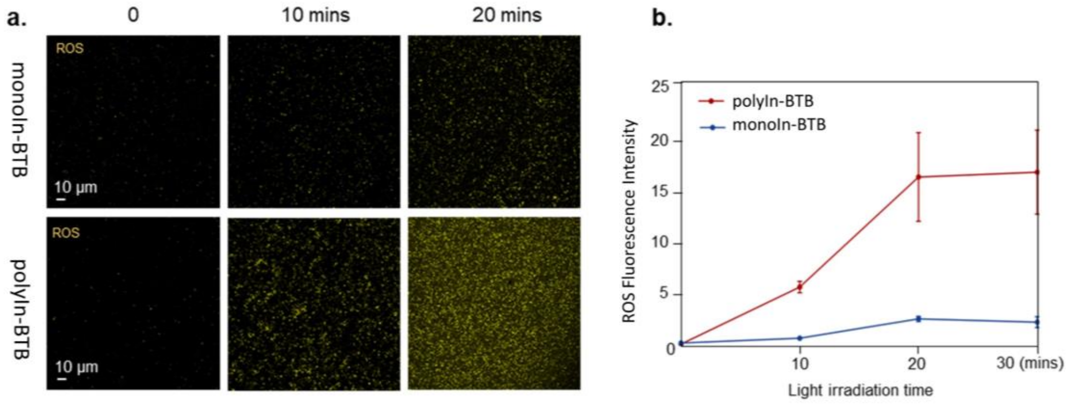

Figure S16. The ROS production efficiency of monoIn-BTB and polyIn-BTB with prolonged light exposure. (a) ROS fluorescent images of *MRS. aureus* under visible light irradiation at 0, 10 mins, and 20 mins after different treatment. (b) The time-dependent ROS concentration (ROS fluorescence intensity under visible light irradiation at 30 mins was calculated by the data in figure 4a and b).

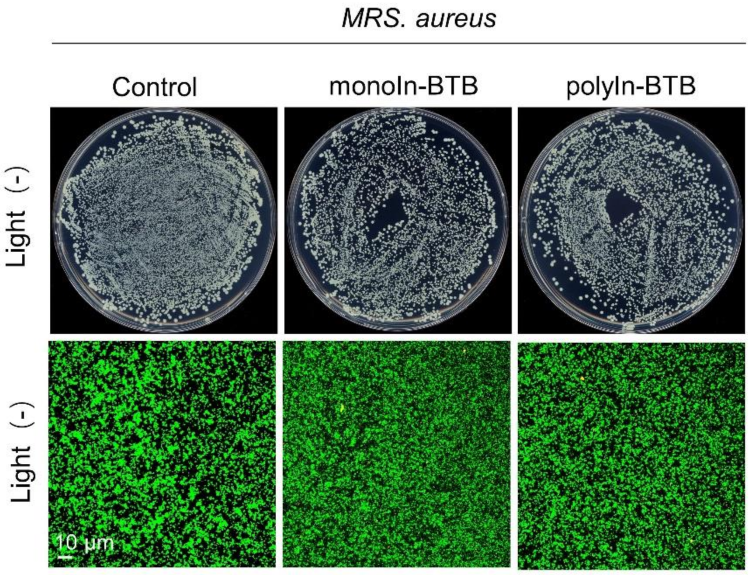

Figure S17. Bacterial colonies and live/dead staining of *MRS. aureus* after different treatments.

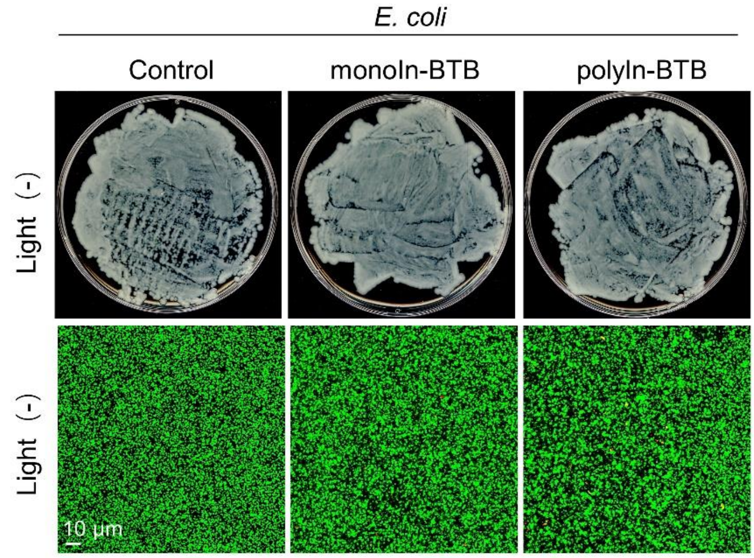

Figure S18. Bacterial colonies and live/dead staining of *E. coli* after different treatments.

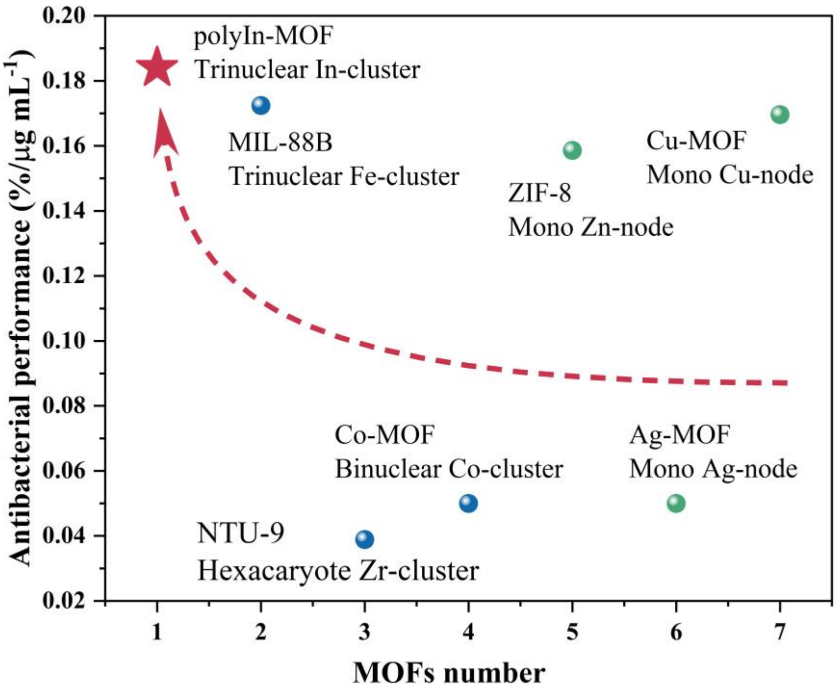

Figure S19. Comparison of antibacterial properties of different MOF materials [3-7]. (Blue represents multicore cluster MOF and green represents single-core MOF)

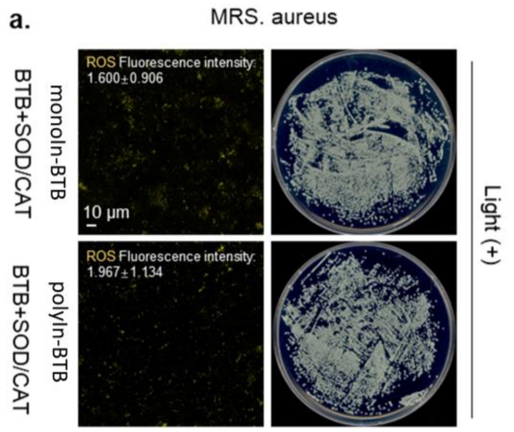

Figure S20. The antibacterial effects of monoIn-BTB and polyIn-BTB are inhibited following ROS scavenging. ROS fluorescent images and bacterial colonies of (a) *MRS. aureus* and (b) *E. coli* after ROS scavenging in In-BTB and polyIn-BTB groups.

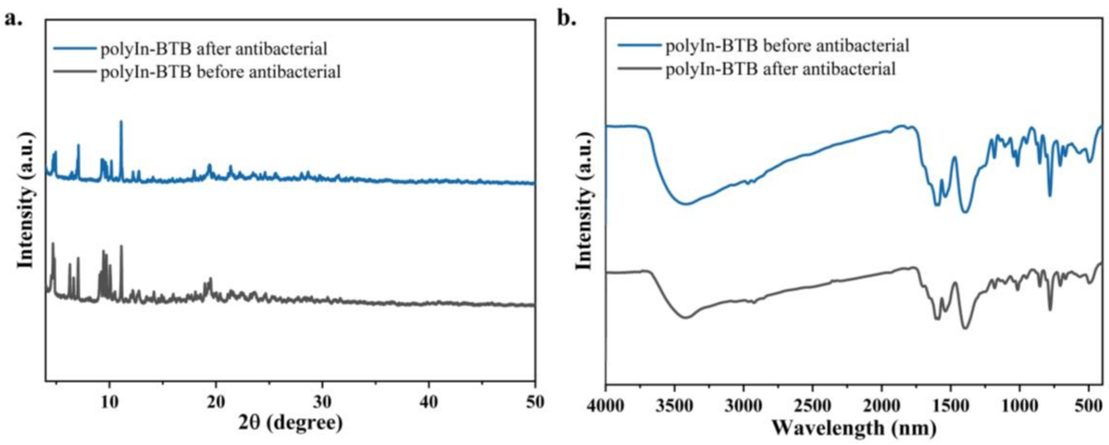

Figure S21. (a) The XRD patterns and (b) FTIR spectra of polyIn-BTB before and after the antibacterial testing.

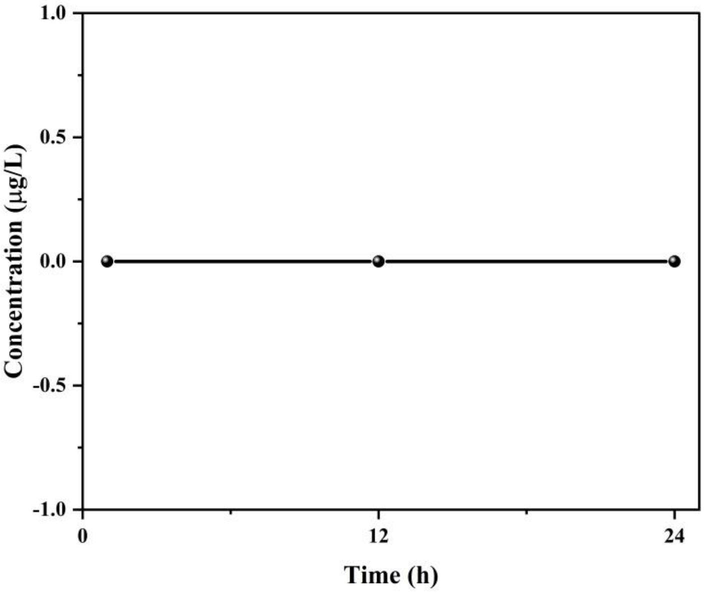

Figure S22. Concentrations of In^{3+} in the culture medium.

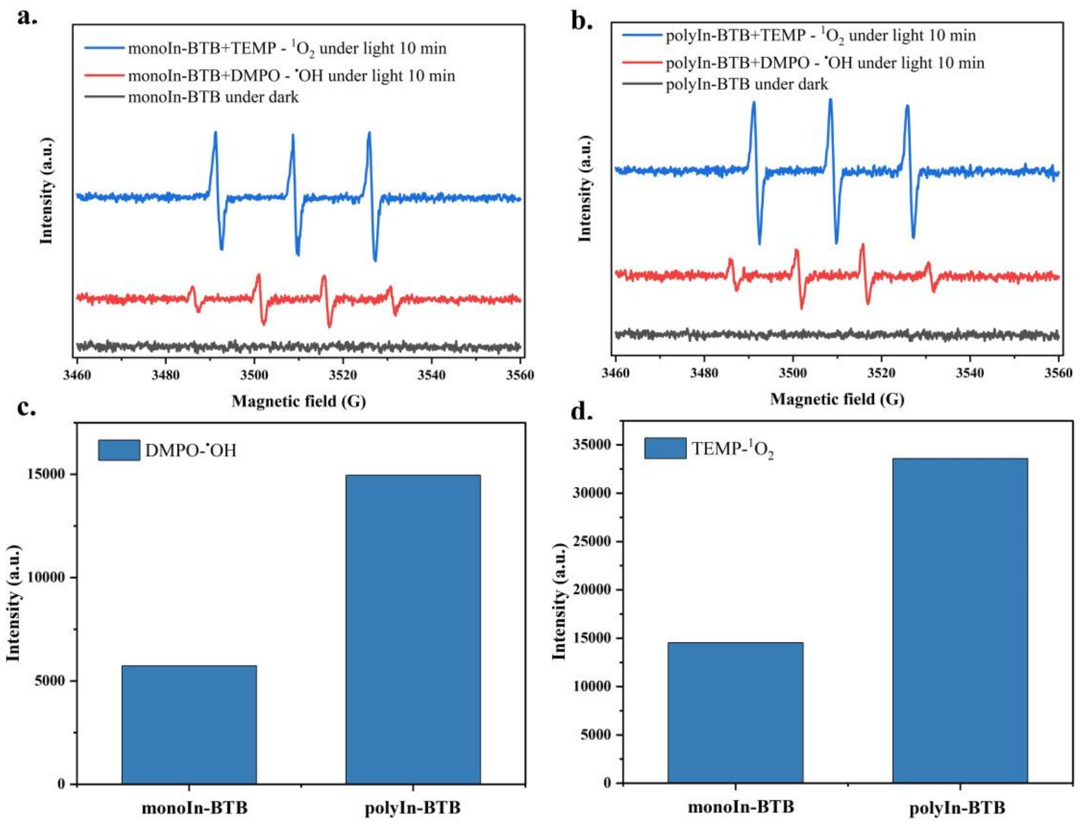

Figure S23. (a) and (b) EPR spectra of $^{\cdot}$OH and $^1O_2$ for (a) monoIn-BTB and (b) polyIn-BTB. (c) The $^{\cdot}$OH intensity of monoIn-BTB and polyIn-BTB. (d) The $^1O_2$ intensity of monoIn-BTB and polyIn-BTB.

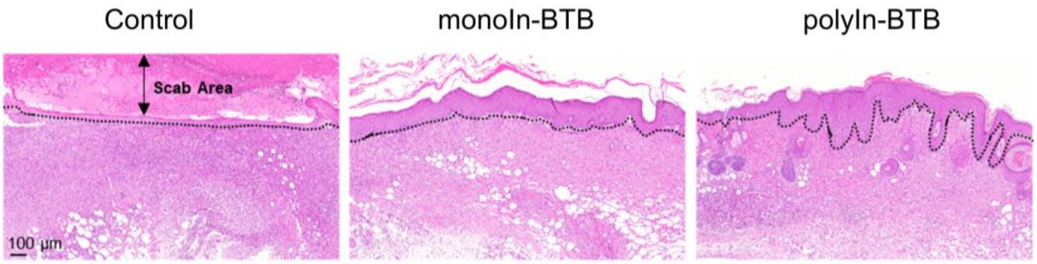

Figure S24. HE staining of the infected skin wounds on day 8 after different treatment. (Dotted line refers to the boundary between the epidermis (above) and dermis (below).

Reference

[1] Z. Li, T. Gao, H. Chu, L. Liao, X. Wang, L. Guo, Y. Xu, W. Zhou, *Appl. Catal. B-Environ. Energy*, **2024**, *358*, 124370.
[2] C. Wei, Y. M. Sun, G. G. Scherer, A. C. Fisher, M. Sherburne, J. W. Ager, Z. C. J. Xu, J. Am. Chem. Soc. **2020**, *142*, 7765.
[3] De Hao, J Li, X Zhou, Y Li, Z Zhao, Rui Zhou, *Small*, **2024**, *20*, 2305943.
[4] D Han, Y Han, J Lia, X Liu, K W K Yeung, Y Zheng,Z Cui, X Yang, Y Liang, Z Li, S Zhu, X Yuan,X Feng, C Yang, S Wu,*Appl. Catal. B-Environ. Energy*, **2020**, *261*, 118248.
[5] K Gwon, I Han, S Lee, Y Kim, D N Lee, *ACS Appl. Mater. Interfaces*, **2020**, *12*, 20234−20242.
[6] M D Firouzjaei, A A Shamsabadi, S A Aktij, S. F Seyedpour, M S Gh., A Rahimpour, M R Esfahani,M Ulbricht, M Soroush, *ACS Appl. Mater. Interfaces*, **2018**, *10*, 42967−42978.
